# EGFR mutation-guided use of afatinib, erlotinib and gefitinib for advanced non-small-cell lung cancer in Hong Kong – A cost-effectiveness analysis

**DOI:** 10.1371/journal.pone.0247860

**Published:** 2021-03-01

**Authors:** Joyce H. S. You, William C. S. Cho, Wai-kit Ming, Yu-chung Li, Chung-kong Kwan, Kwok-hung Au, Joseph Siu-kie Au

**Affiliations:** 1 School of Pharmacy, Faculty of Medicine, The Chinese University of Hong Kong, Hong Kong SAR, China; 2 Department of Clinical Oncology, Hospital Authority, Queen Elizabeth Hospital, Hong Kong SAR, China; 3 Hong Kong United Oncology Centre, Hong Kong SAR, China; 4 Department of Oncology, Hospital Authority, United Christian Hospital, Hong Kong SAR, China; 5 Oncology Center, Hong Kong Adventist Hospital, Hong Kong SAR, China; Chang Gung Memorial Hospital and Chang Gung University, Taoyuan, Taiwan, TAIWAN

## Abstract

**Introduction:**

Tyrosine kinase inhibitors (TKIs) therapy targets at epidermal growth factor receptor (EGFR) gene mutations in non-small-cell lung cancer (NSCLC). We aimed to compare the EGFR mutation-guided target therapy versus empirical chemotherapy for first-line treatment of advanced NSCLC in the public healthcare setting of Hong Kong.

**Methods:**

A Markov model was designed to simulate outcomes of a hypothetical cohort of advanced (stage IIIB/IV) NSCLC adult patients with un-tested EGFR-sensitizing mutation status. Four treatment strategies were evaluated: Empirical first-line chemotherapy with cisplatin-pemetrexed (empirical chemotherapy group), and EGFR mutation-guided use of a TKI (afatinib, erlotinib, and gefitinib). Model outcome measures were direct medical cost, progression-free survival, overall survival, and quality-adjusted life-years (QALYs). Incremental cost per QALY gained (ICER) was estimated. Sensitivity analyses were performed to examine robustness of model results.

**Results:**

Empirical chemotherapy and EGFR mutation-guided gefitinib gained lower QALYs at higher costs than the erlotinib group. Comparing with EGFR mutation-guided erlotinib, the afatinib strategy gained additional QALYs with ICER (540,633 USD/QALY). In 10,000 Monte Carlo simulations for probabilistic sensitivity analysis, EGFR mutation-guided afatinib, erlotinib, gefitinib and empirical chemotherapy were preferred strategy in 0%, 98%, 0% and 2% of time at willingness-to-pay (WTP) 47,812 USD/QALY (1x gross domestic product (GDP) per capita), and in 30%, 68%, 2% and 0% of time at WTP 143,436 USD/QALY (3x GDP per capita), respectively.

**Conclusions:**

EGFR mutation-guided erlotinib appears to be the cost-effective strategy from the perspective of Hong Kong public healthcare provider over a broad range of WTP.

## Introduction

Lung cancer is the second most common cancer, with highest mortality (crude rate 52.6 per 100,000 persons) among the top 10 cancers in Hong Kong [1]. Almost 85%-90% of lung cancers are classified as non-small-cell lung cancer (NSCLC), and adenocarcinoma is the most common (78%) subtype of NSCLC. Approximately 80% of NSCLC are diagnosed in advanced stage (IIIB/IV). Standard platinum-based chemotherapy for advanced NSCLC modestly lengthens survival by a few months [2].

Epidermal growth factor receptor (EGFR) gene mutations are actionable targets in NSCLC [3]. These mutations are significantly correlated with treatment response to tyrosine kinase inhibitors (TKIs) therapy. EGFR gene mutations are only found in 10%-15% of Caucasian patients, but are more frequently (>50%) observed in Asian population. In Hong Kong, the reported incidence of EGFR mutation-positive was 47% in patients with NSCLC of adenocarcinoma histology [2, 4]. Multiple clinical trials and meta-analysis showed first-line TKIs (afatinib, erlotinib, and gefitinib) to prolong progression-free survival and delay disease progression in EGFR mutation-positive patients [5–8]. Health economics studies on first-line TKIs for advanced NSCLC treatment found that the incremental cost per quality-adjusted life-year gained (ICER) by TKI was high (USD100,000–150,000 per QALY) in patients with low prevalence of EGFR mutation (9.5%-15%) [9, 10]. Low ICER (<USD50,000 per QALY) or QALY gain with cost-saving were reported in patient population with high EGFR mutation prevalence (30%-60%) [11, 12]. In order to assist the informed decision-making process on TKI selection for NSCLC treatment, the present study aimed to compare the EGFR mutation-guided use of afatinib, erlotinib, and gefitinib versus empirical chemotherapy for first-line treatment of advanced NSCLC in the public healthcare setting of Hong Kong.

## Methods

### Model design

**Fig 1:**
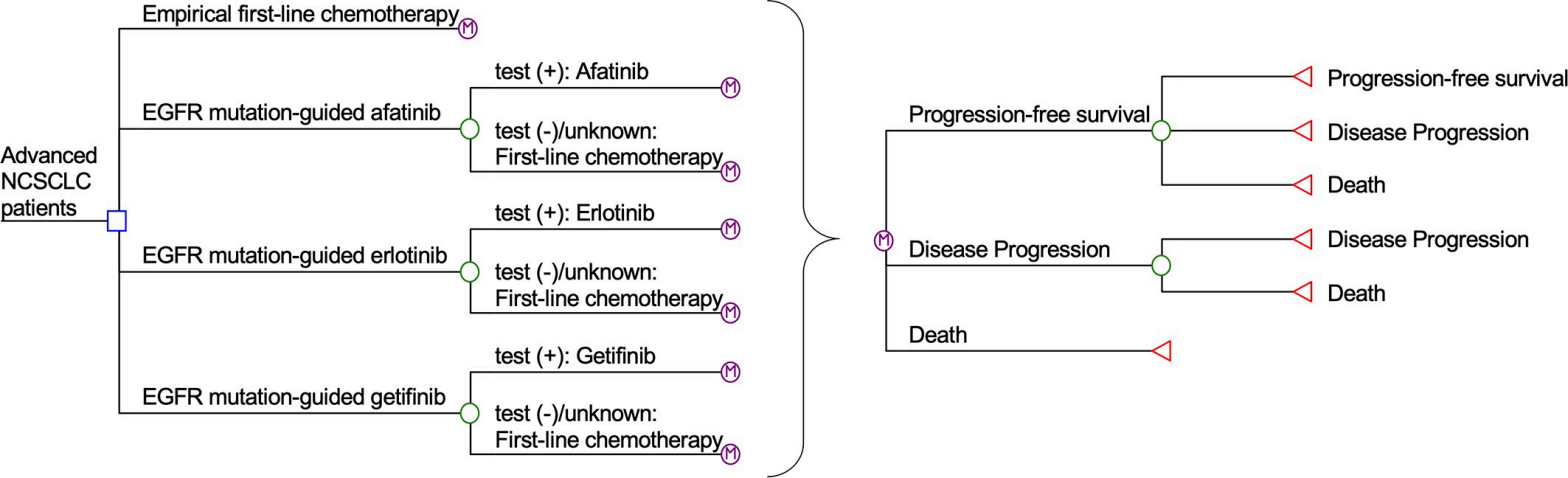
Simplified Markov model of empirical chemotherapy, EGFR mutation-guided afatinib, erlotinib, and gefitinib for treatment of advanced NSCLC. EGFR: epidermal growth factor receptor; NSCLC: non-small-cell lung cancer.

A Markov model was designed to simulate outcomes of a hypothetical cohort of advanced (stage IIIB/IV) NSCLC adult patients with untested EGFR-sensitizing mutation status (**Fig 1**). Markov model is an analytical framework in which the hypothetical patients proceed to mutually exclusive health states in the next model cycle according to transition probabilities, with costs and health outcomes aggregated over successive cycles. Four treatment strategies were evaluated in the present model: Empirical first-line chemotherapy with cisplatin-pemetrexed (empirical chemotherapy group) for all patients, and EGFR mutation-guided use of afatinib, erlotinib, and gefitinib. The model time horizon was 10 years (with monthly cycle) to allow adequate time for estimation of the lifelong outcome measures, including direct medical cost, progression-free survival, life-years and QALYs gained by each treatment strategy.

Major health states in the Markov model included: Progression-free survival, disease progression and death. All patients entered the model at the health status of progression-free survival. The patients proceeded through health statuses in each monthly cycle according to probability inputs. In the empirical treatment group, the patients received cisplatin-pemetrexed for the up to six 21-day cycles [13]. In the EGFR-guided groups, patients who were tested positive for EGFR mutation were treated with gefitinib, erlotinib, or afatinib until disease progression occurred [5–7]. Patients with negative or undetermined EGFR-mutation testing results were treated with first-line chemotherapy. Palliative care, including palliative chemotherapy with pemetrexed and best supportive care, was provided to control symptoms for patients with disease progression [3].

### Clinical inputs

All model inputs are listed in **Table 1**. Literature search on MEDLINE over the period 2000–2020 was performed using keywords including “advanced non-small-cell lung cancer”, “NSCLC”, “EGFR mutation”, “overall survival”, “progression-free survival”, “first-line treatment”, “first-line chemotherapy”, “gefitinib”, “erlotinib”, and “afatinib”. The selection criteria of clinical trials were: (1) reports in English language; (2) adult patients with stage IIIB/IV NSCLC; and (3) progression-free survival, overall survival or adverse event rates were reported. Preferred studies were meta-analyses or randomized controlled trials. When multiple randomized trials were available for the same model input, the weighted average was used as the base-case value and the high/low values formed as the upper/lower limits for sensitivity analysis.

**Table 1:** Model inputs.

Parameters	Base-case value	Range of sensitivity analysis	Distribution	Reference
**Clinical inputs**				
EGFR mutation prevalence	50.1%	42.7%-58.8%	Beta	Local
First-line chemotherapy (cisplatin-pemetrexed) Weibull parameters				
Progression-free survival				
Scale parameter (λ)	0.0572	Fixed	-	[13]
Shape parameter (γ)	1.2883	Fixed	-	[13]
Overall survival				
Scale parameter (λ)	0.0057	Fixed	-	[14]
Shape parameter (γ)	1.4499	Fixed	-	[14]
Odds ratios versus first-line chemotherapy			Triangular	
Progression-free survival				
Afatinib	8.31	3.19–23.21		[16]
Ertotinib	6.57	2.74–15.1		[16]
Gefitinib	6.12	2.87–13.5		[16]
Overall survival				
Afatinib	1.11	0.62–1.96		[16]
Ertotinib	1.08	0.67–1.76		[16]
Gefitinib	0.80	0.50–1.27		[16]
**Utility inputs**				
Utility value			Triangular	
Progression-free survival	0.80	0.77–0.84		[18]
Disease progression	0.56	0.47–0.66		[18]
Disutility value			Triangular	
Nausea/vomiting	-0.048	-(0.0.016–0.80)		[17]
Diarrhea	-0.047	-(0.016–0.077)		[17]
Rash	-0.032	-(0.01–0.05)		[17]
Anemia	-0.07	-(0.04–0.1)		[17]
Neutropenic fever	-0.09	-(0.06–0.12)	Triangular	[17]
Incidence of serious adverse event				
Afatinib			Beta	
Nausea/vomiting	0.80%	0.64%-0.96%		[6]
Diarrhea	5.4%	4.3%-6.5%		[6]
Rash	14.2%	11.4%-17.0%		[6]
Anemia	0.40%	0.3%-0.5%		[6]
Neutropenic fever	0.40%	0.3%-0.5%		[6]
Erlotinib			Beta	
Nausea/vomiting	0.0%	0.0%-0.01%		[7]
Diarrhea	1.0%	0.8%-1.2%		[7]
Rash	2.0%	1.6%-2.4%		[7]
Anemia	0%	0.0%-0.01%		[7]
Neutropenic fever	0%	0.0%-0.01%		[7]
Gefitinib			Beta	
Nausea/vomiting	0.3%	0.2%-0.4%		[5]
Diarrhea	3.8%	3.0%-4.6%		[5]
Rash	3.1%	2.5%-3.7%		[5]
Anemia	2.2%	1.8%-2.6%		[5]
Neutropenic fever	0.2%	0.1%-0.24%		[5]
Cisplatin-pemetrexed			Beta	
Nausea/vomiting	3.6%	2.9%-4.3%		[13]
Diarrhea	0.0%	0.0%-0.01%		[13]
Rash	0.0%	0.0%-0.01%		[13]
Anemia	6.3%	5.0%-7.6%		[13]
Neutropenic fever	18.0%	14.4%-21.6%		[13]
**Cost inputs (USD)**	Median	IQR	Triangular	Local cohort
EGFR (per test)	324	284–432		
Cost per month (including drug and non-drug costs)				
Progression-free survival state				
Afatinib therapy	1870	1741–2749		
Erlotinib therapy	447	318–1326		
Gefitinib therapy	1341	1212–2220		
Cisplatin-pemetrexed therapy	2374	1159–4410		
Off-chemotherapy follow up	152	122–183		
Disease progression state				
Palliative care	493	233–1013		
Cost of serious adverse event per episode				
Nausea/vomiting	160	159–177		
Diarrhea	153	152–153		
Rash	155	154–155		
Anemia	654	653–693		
Neutropenic fever	4479	2267–4729		

EGFR: Epidermal growth factor receptor.

The prevalence of EGFR mutation-positive in advanced NSCLC Hong Kong patients (50.1%; range 42.7%-58.8%) was approximated from 5-year pathological data (2013–2017) of the Queen Elizabeth Hospital, largest public hospital (1,844 beds) of the Hospital Authority. The probabilities of progression-free survival and overall survival among patients treated with cisplatin-pemetrexed were estimated from the Kaplan-Meier survival curves of progression-free survival and overall survival, respectively. Data were first extracted from the corresponding Kaplan-Meier survival curves of cisplatin-pemetrexed treatment group in prospective clinical trials on EGFR mutation-positive NSCLC treatment [13, 14]. Weibull curves were used to fit to the extracted data and to estimate scale parameter (λ) and shape parameter (γ). The probability of transition at the time point of *t* with cisplatin-pemetrexed treatment was estimated by the following formula [15]:

$$P(t)=1-\text{exp}[\gamma {\left(t-1\right)}^{\gamma }-\lambda t^{\gamma }]$$

A network meta-analysis, including 16 phase III randomized trials with 2,962 advanced NSCLC patients, integrated the treatment outcomes of gefitinib, erlotinib, afatinib and first-line chemotherapy [16]. The odds ratios (and 95%CI) for progression-free survival and overall survival with each TKI (gefitinib, erlotinib, afatinib) versus first-line chemotherapy reported in the network meta-analysis were adopted in the present model. The monthly probabilities of each TKI to remain in progression-free survival and in overall survival were estimated by the corresponding odds ratio and the monthly probability of event with cisplatin-pemetrexed.

### Utility inputs

The utility inputs were shown in **Table 1**. The QALYs expected by each subject was estimated from cumulative subject-time spent in a heath state and the health state-specific utility value. The health states included progression-free survival, disease progression and death. The utility of the progression-free survival was further adjusted with disutility of treatment-related serious adverse events (SAEs) (≥Grade 3). The utility and disutility values were retrieved from literature on advanced NSCLC-related quality of life and health economic analysis [17, 18]. The incidence of SAEs with afatinib, erlotinib, gefitinib, and cisplatin-pemetrexed were adopted from the findings of randomized clinical trials on treatment of advanced NSCLC in Asian patients [5–7, 13]. The QALY gained over the model time horizon was discounted by an annual rate of 3%.

### Cost inputs

The health economic analysis was conducted on direct medical costs from the perspective of Hong Kong public healthcare provider. Direct costs included biopsy-based EGFR mutation testing (for TKI arms), cost per month in progression-free survival state, cost per month for palliative care in disease progression state, and cost of managing SAEs. The healthcare resource utilization during progression-free survival and disease progression were estimated retrospectively at the Hospital Authority of Hong Kong. The Hospital Authority is the sole public healthcare provider in Hong Kong. Medical record review was conducted for patients aged ≥18 years with diagnosis of advanced (stage IIIB/IV) NSCLC (n = 400; 59% male; mean age 67±12 years) who were treated with first-line chemotherapy or TKI in 2013 to 2017 at two general hospitals (Queen Elizabeth Hospital and United Christian Hospital) of the Hospital Authority (Kowloon Central cluster and Kowloon East cluster, respectively). The study protocol was approved by the Research Ethics Committee for Kowloon Central / Kowloon East cluster. Healthcare resource utilization (including usage of drugs, clinic visit, hospitalization and diagnostic testing) were collected to estimate monthly direct medical costs for progression-free survival state and disease progression state, and management cost per episode of treatment-related SAEs, using unit cost of 2020. The costs accumulated over the 10-year model timeframe were discounted with an annual rate of 3%.

### Cost-effectiveness and sensitivity analyses

Cost-effectiveness and sensitivity analyses were conducted by TreeAge Pro 2020 (TreeAge Software Inc., Williamstown, MA) and Microsoft Excel 2016 (Microsoft Corporation, Redmond, WA, USA). Base-case analysis compared the expected direct medical cost and QALYs of each EGFR mutation-guided TKI therapy with the empirical chemotherapy, assuming 100% TKI compliance as indicated by mutation test results. A treatment strategy was dominated when it gained lower QALYs at higher cost than another option, and the dominated option was eliminated from further cost-effectiveness analysis. If a treatment strategy gained more QALYs at higher cost than another alternative, incremental cost per QALY gained (ICER) of the more effective strategy was calculated: Δcost/ΔQALYs. The World Health Organization (WHO) recommended that ICER less than 1× gross domestic product (GDP) per capita to be highly cost-effective and less than 3× GDP per capita to be cost-effective [19]. The GDP per capita of Hong Kong was USD47,812 in 2019 [20] and USD143,436 (3× GDP per capita) was adopted as the willingness-to-pay (WTP) threshold in the base-case analysis. A treatment alternative was preferred if (1) it was effective in saving QALYs at lower cost, or (2) it was effective in saving QALYs at higher cost and the ICER was less than the WTP threshold.

Sensitivity analyses were performed by one-way sensitivity analysis on all model inputs to identify threshold values of influential factors. The probabilistic sensitivity analysis was performed using Monte Carlo simulation. The direct cost and QALYs of each study arm were recalculated 10,000 times by randomly drawing each of the model input from the probability distribution specified in **Table 1**. The probability of each strategy to be accepted as cost-effective was determined over a range of WTP from 0–200,000 USD/QALY in the acceptability curves. The incremental costs and incremental QALYs of EGFR mutation-guided TKI versus chemotherapy comparator were presented in scatter plots.

## Results

### Base-case analysis

Expected direct medical costs, progression-free survival months, overall-survival months, and quality-adjusted life-years (QALYs) are shown in **Table 2**. In the base-case analysis of incremental costs and QALYs (**Table 3**), EGFR mutation-guided use of all three TKIs (afatinib, erlotinib, gefitinib) gained higher QALYs than empirical chemotherapy with cisplatin-pemetrexed. When comparing to empirical chemotherapy (as common comparator), EGFR mutation-guided erlotinib gained higher QALYs with cost-saving and the ICER of afatinib was lower than WTP threshold (143,436 USD/QALY). Both strategies of EGFR mutation-guided erlotinib and afatinib were accepted as cost-effective versus empirical chemotherapy. EGFR-guided gefitinib gained higher QALY than empirical chemotherapy at an ICER (428,208 USD/QALY) exceeding WTP, and was therefore not accepted as cost-effective.

**Table 2:** Expected direct medical cost, progression-free survival (PFS) months, overall-survival (OS) months, and quality-adjusted life-years (QALYs) in base-case analysis.

Strategy	Direct costs (USD)	PFS (months)	OS (months)	QALY
Empirical chemotherapy (cisplatin-pemetrexed)	21,458	8.67	33.30	1.6358
EGFR mutation-guided erlotinib	18,724	15.78	34.18	1.8072
EGFR mutation-guided gefitinib	25,997	14.30	30.98	1.6464
EGFR mutation-guided afatinib	35,808	16.79	34.50	1.8388

**Table 3:** Base-case analysis of increment cost, incremental QALYs and incremental cost per QALY gained (ICER).

	versus empirical chemotherapy			versus the next less costly strategy		
Strategy	Incremental cost (USD)	Incremental QALYs	ICER (USD per QALY)	Incremental cost (USD)	Incremental QALYs	ICER (USD per QALY)
Empirical chemotherapy (cisplatin-pemetrexed)	-	-	-	2,734	-0.1714	Dominated* by EGFR mutation-guided erlotinib
EGFR mutation-guided erlotinib	-2,734	0.1714	Dominating empirical therapy	-	-	-
EGFR mutation-guided gefitinib	4,539	0.0106	428,208	7,273	-0.1608	Dominated* by EGFR mutation-guided erlotinib
EGFR mutation-guided afatinib	14,350	0.2030	70,690	17,084	0.0316	540,633

*Dominated strategy are eliminated from ICER analysis.

QALY: quality-adjusted life-year.

Two strategies (empirical chemotherapy and EGFR mutation-guided gefitinib) gained lower QALYs at higher costs than the erlotinib group, and were therefore dominated by the erlotinib strategy (and the dominated arms were eliminated). Comparing with EGFR mutation-guided erlotinib (as the-less-costly strategy), the afatinib strategy gained additional QALYs at higher cost by an ICER of 540,633 USD/QALY (>WTP threshold), and was not accepted to be cost-effective. EGFR mutation-guided erlotinib was the only cost-effective strategy versus both the common comparator and the next-less-costly strategy in the base-case analysis.

### Sensitivity analysis

One-way sensitivity analysis found that the base-case results were robust to the variation of all model inputs and no threshold value was identified. The EGFR mutation-guided afatinib strategy gained the highest QALYs with the ICER (versus EGFR mutation-guided erlotinib) exceeding the WTP threshold. The monthly cost of afatinib therapy was further examined in an extended one-way sensitivity analysis from the base-case value (USD1870) to lower limit (USD200), for identification of a threshold value. The ICER of EGFR mutation-guided afatinib became lower than the WTP threshold (and accepted as cost-effective) when the monthly cost of afatinib therapy was lower than USD818. The compliance to use TKI (base-case value 100%, range 50%-100%) among EGFR mutation-positive patients was also added to one-way sensitivity analysis, and no threshold value was identified.

**Fig 2:**
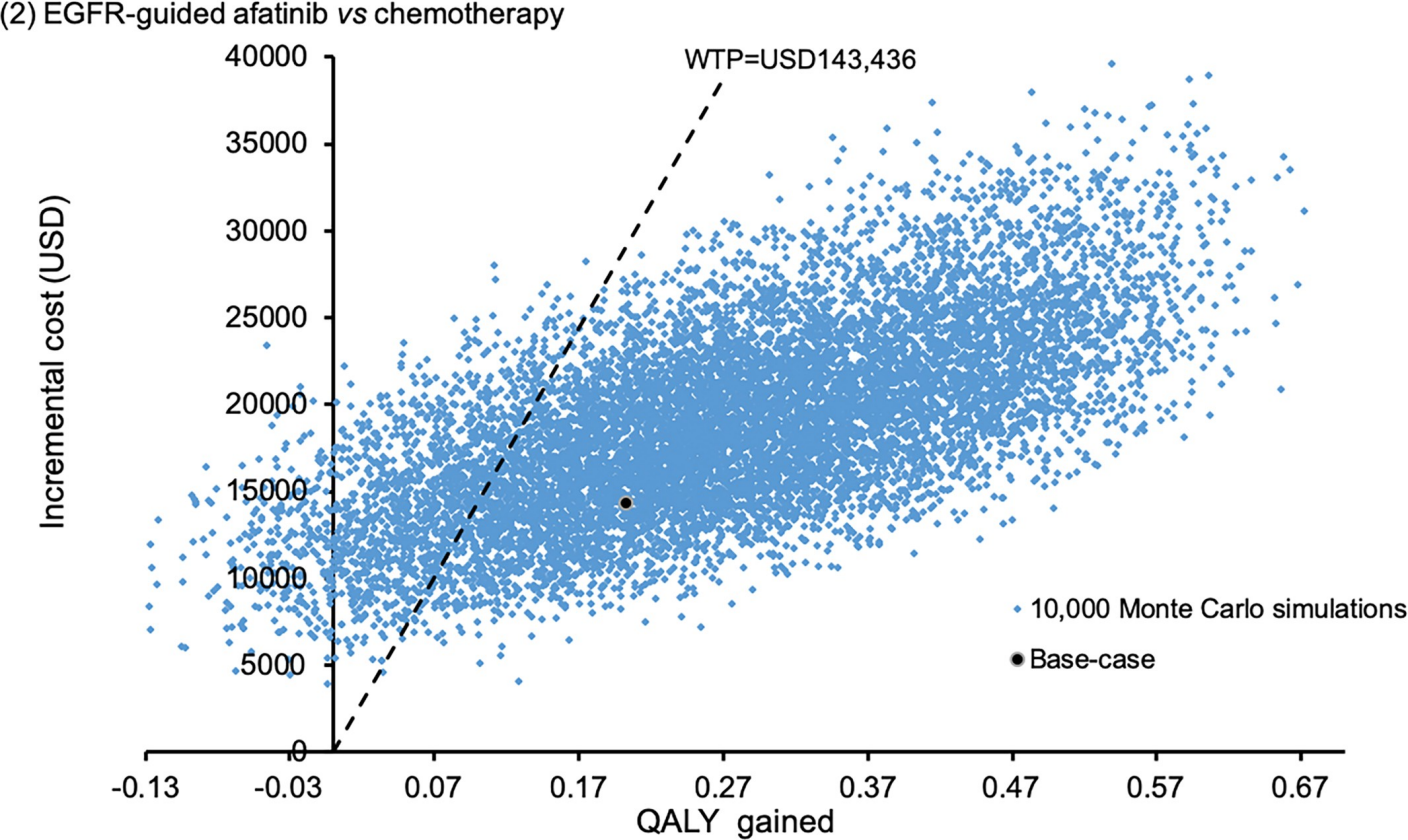
Scatter plot of incremental cost against QALY gained by EGFR mutation-guided afatinib versus empirical chemotherapy. QALY: quality-adjusted life-year; WTP: willingness-to-pay.

**Fig 3:**
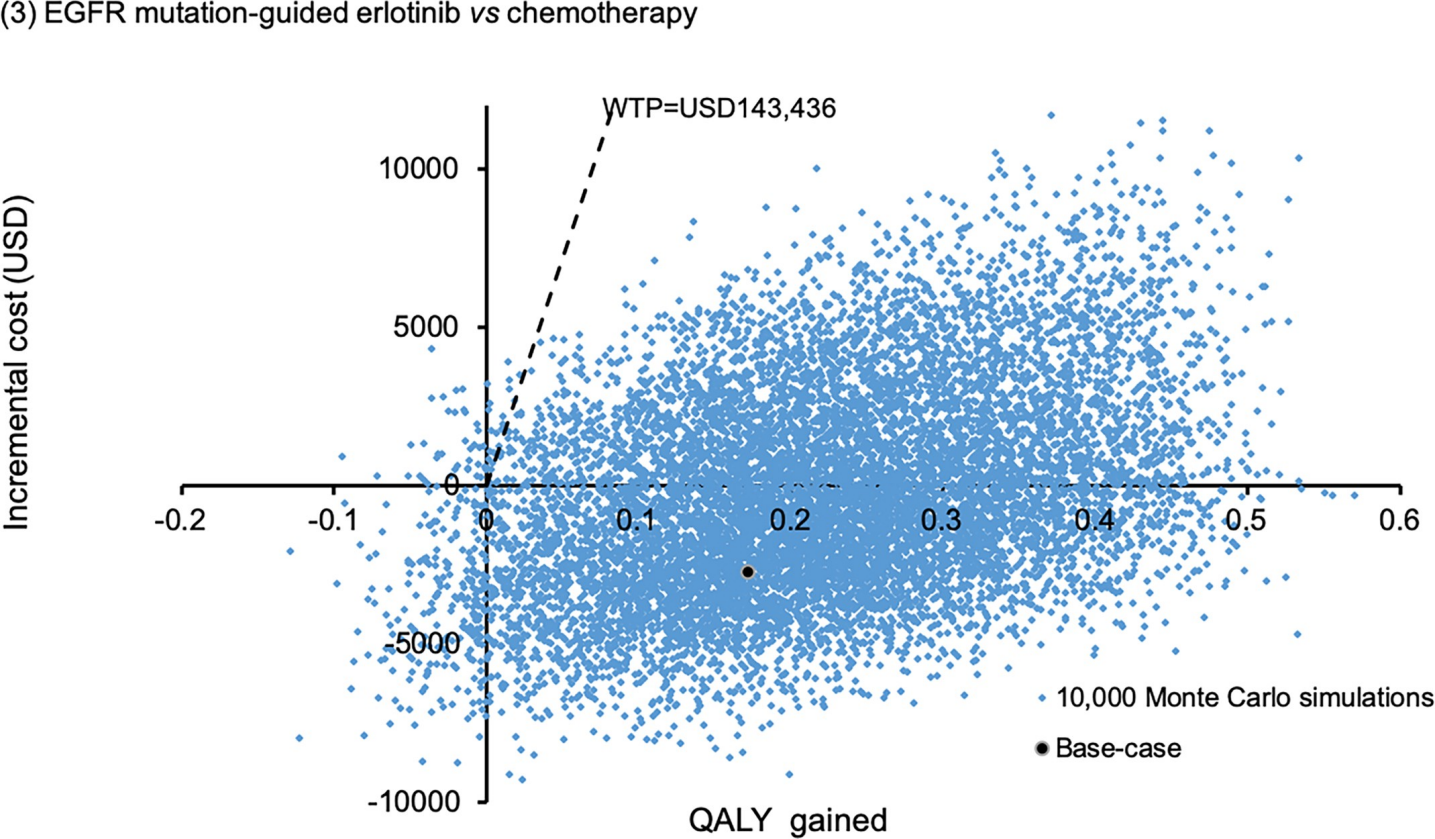
Scatter plot of incremental cost against QALY gained by EGFR mutation-guided erlotinib versus empirical chemotherapy. QALY: quality-adjusted life-year; WTP: willingness-to-pay.

**Fig 4:**
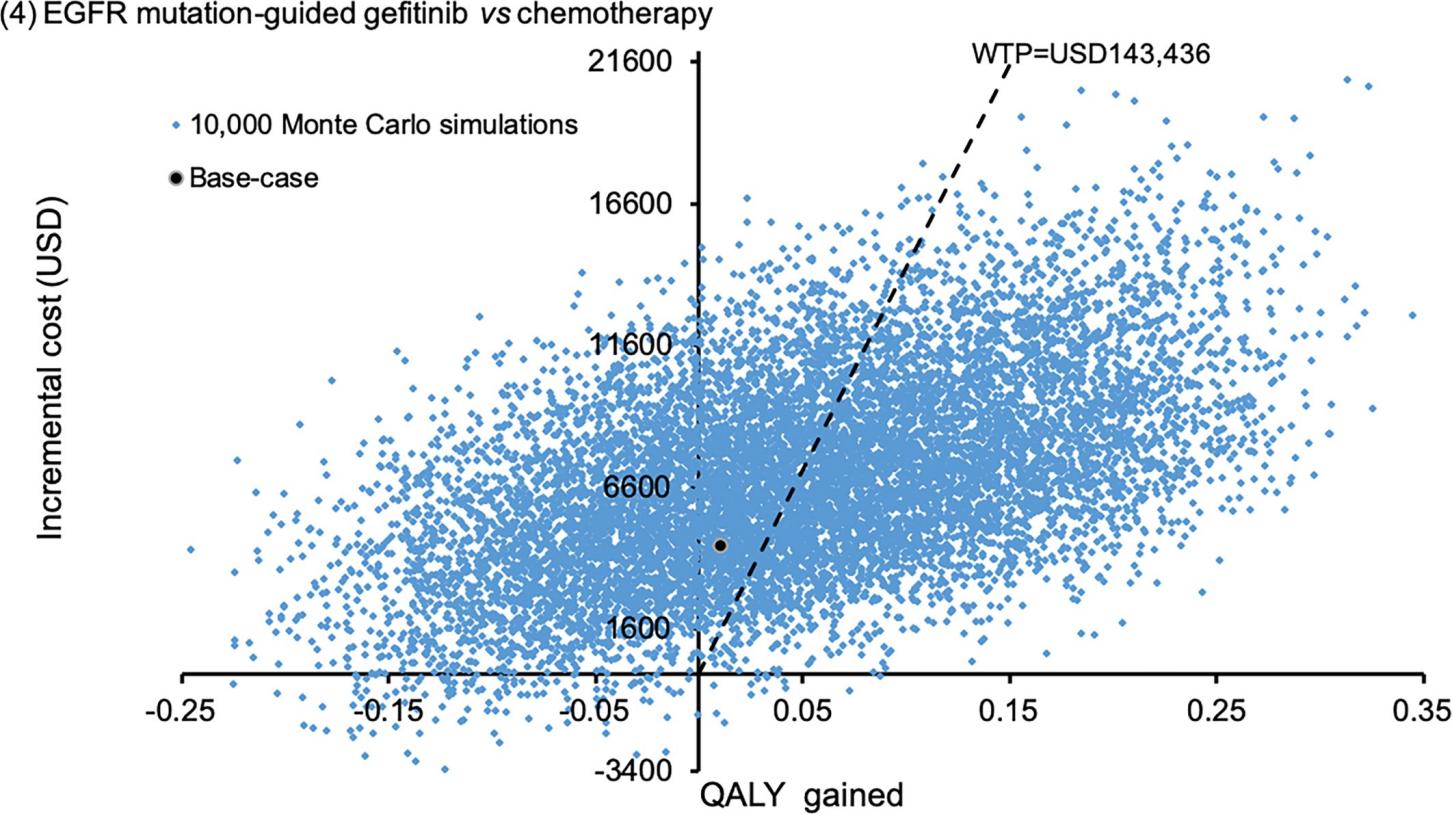
Scatter plot of incremental cost against QALY gained by EGFR mutation-guided gefitinib versus empirical chemotherapy. QALY: quality-adjusted life-year; WTP: willingness-to-pay.

The probabilistic sensitivity analysis was performed by 10,000 Monte Carlo simulations. Comparing to empirical chemotherapy, the EGFR mutation-guided afatinib gained additional 0.2727 QALYs (95%CI 0.2699–0.2756; p<0.01) by incremental cost of USD18,838 (95%CI 18,733–18,944; p<0.01); erlotinib gained additional 0.2217 QALYs (95%CI 0.2194–0.2241; p<0.01) with cost saving of USD281 (95%CI 218–345; p<0.01); gefitinib gained additional 0.0487 QALYs (95%CI 0.0468–0.0507; p<0.01) by incremental cost of USD6,937 (95%CI 6,868–7,005; p<0.01). **Figs 2–4** showed the scatter plots of the increment cost and QALYs gained by each EGFR mutation-guided TKI versus empirical chemotherapy. EGFR mutation-guided afatinib, erlotinib and gefitinib were accepted as cost-effective (with QALY gains and ICER<WTP) versus empirical chemotherapy in 86.2%, 97.2%, and 49.5% of the 10,000 simulations.

**Fig 5:**
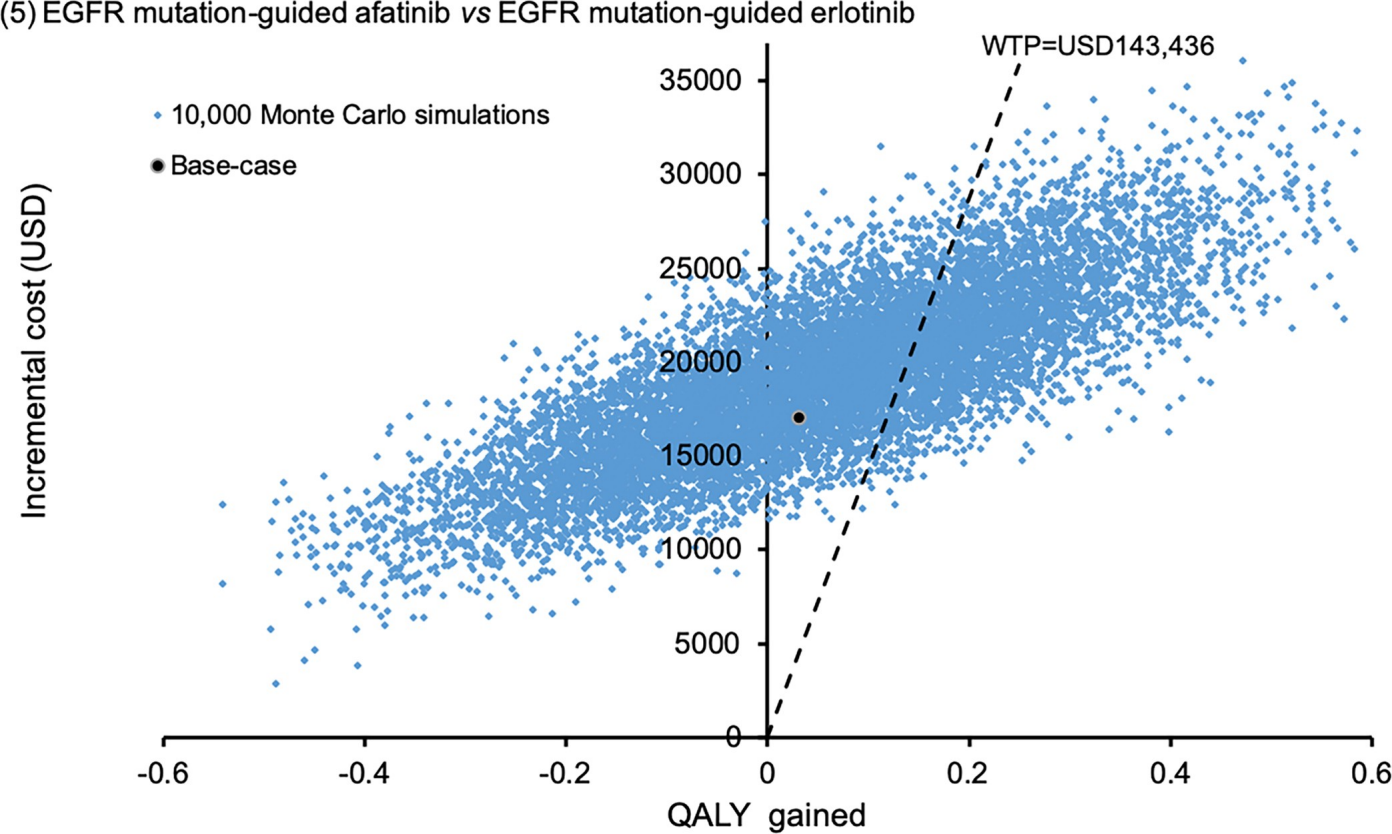
Scatter plot of incremental cost against QALY gained EGFR mutation-guided afatinib versus EGFR mutation-guided erlotinib. QALY: quality-adjusted life-year; WTP: willingness-to-pay.

Comparing EGFR mutation-guided afatinib versus erlotinib, afatinib gained additional 0.0510 QALYs (95%CI 0.0474–0.0546; p<0.01) by incremental cost of USD19,120 (95%CI 19,030–19,209). **Fig 5** showed the scatter plots of EGFR mutation-guided afatinib versus erlotinib. The afatinib strategy was accepted as cost-effective (with QALY gains and ICER<WTP) in 31.2% of the time comparing to erlotinib.

**Fig 6:**
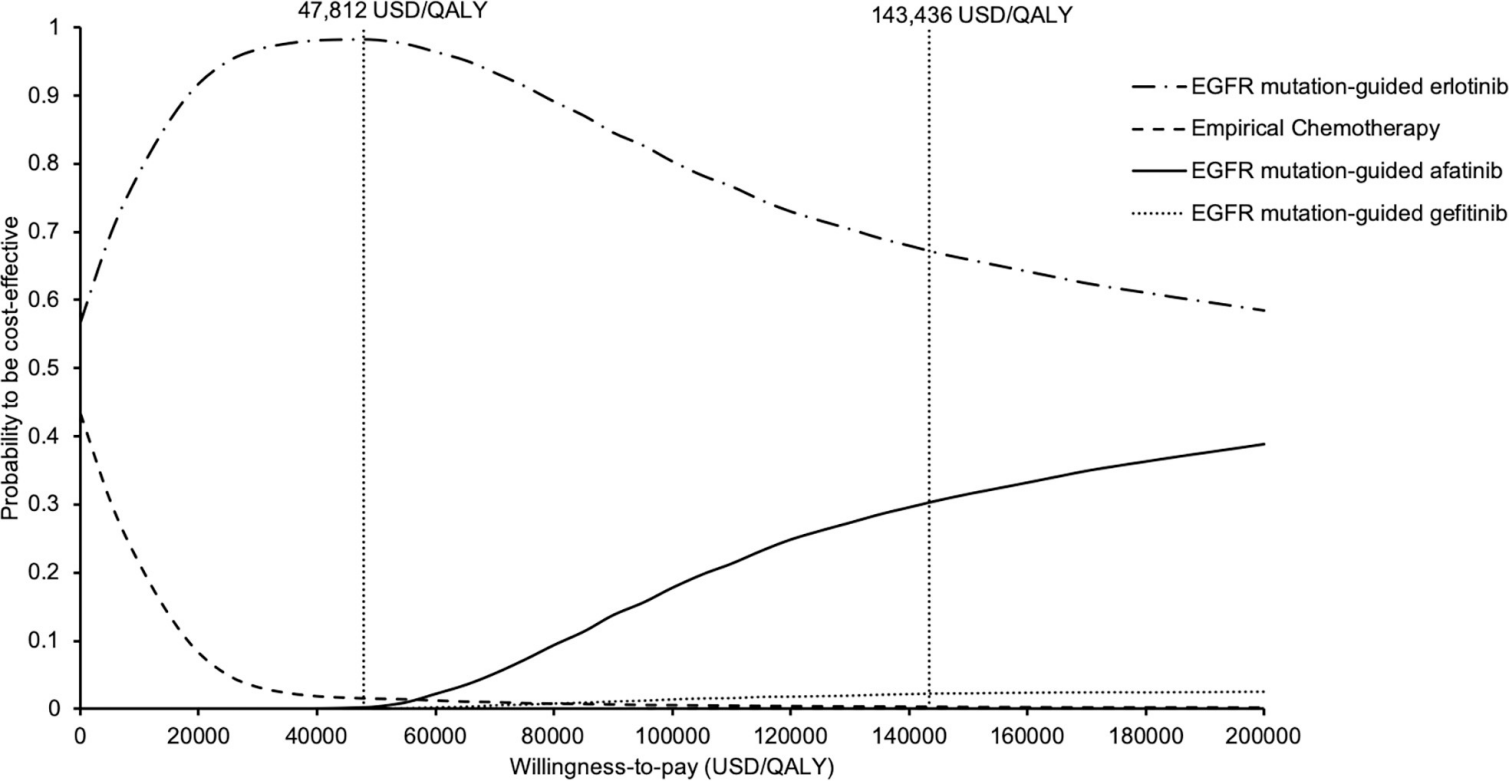
Acceptability curves of empirical chemotherapy, EGFR mutation-guided afatinib, erlotinib, and gefitinib for treatment of advanced NSCLC to be cost-effective against willingness-to-pay. EGFR: epidermal growth factor receptor; NSCLC: non-small-cell lung cancer.

To examine the acceptability of four treatment arms simultaneously, the probabilities of each treatment strategy to be accepted as cost-effective were shown in the acceptability curves over a wide range of WTP (0–200,000 USD/QALY) (**Fig 6**). EGFR mutation-guided afatinib, erlotinib, gefitinib and empirical chemotherapy were accepted to be preferred strategy in 0%, 98%, 0% and 2% of time at WTP 47,812 USD/QALY (1x GDP per capita), and in 30%, 68%, 2% and 0% of time at WTP 143,436 USD/QALY (3x GDP per capita), respectively.

## Discussion

The present findings showed EGFR mutation-guided use of afatinib, erlotinib and gefitinib to gain higher QALYs than empirical chemotherapy of cisplatin-pemetrexed (without EGFR mutation testing). EGFR mutation-guided afatinib and gefitinib were more costly than empirical chemotherapy, whilst EGFR mutation-guided erlotinib was the cost-saving strategy. The ICER analysis of the base-case costs and QALYs showed the EGFR mutation-guided erlotinib to be preferred strategy from the perspective of public healthcare provider in Hong Kong. The probabilistic sensitivity analyses supported the base-case findings to be highly robust that the EGFR mutation-guided erlotinib gained higher QALYs with cost saving when compared with empirical chemotherapy.

EGFR mutation-guided afatinib therapy gained the highest QALYs in the present findings, yet the probability to be cost-effective as indicated in the acceptability curves remained lower than the erlotinib strategy throughout a wide range of WTP (0–200,000 USD/QALY). The extended one-way sensitivity analysis on monthly cost of afatinib therapy showed that a 56% reduction from USD1870 to USD818 per month would achieve an ICER less than the WTP threshold of 143,436 USD/QALY.

A recent cost-effectiveness analysis of afatinib, gefitinib, erlotinib and pemetrexed-based chemotherapy as first-line treatment for EGFR mutation-positive NSCLC patients was reported in China [21]. The QALY gain was highest with afatinib, followed by erlotinib, gefitinib and pemetrexed-cisplatin, and the afatinib was accepted as cost-effective versus both chemotherapy and erlotinib. A health economic analysis comparing afatinib versus gefitinib for first-line treatment for EGFR-mutated NSCLC also reported higher QALY gained at additional cost by afatinib versus gefitinib in China [22]. Our findings are consistent with the reported cost-effectiveness analyses in China that the afatinib strategy gained higher QALYs than chemotherapy, gefitinib and erlotinib. The present analysis also demonstrated a high likelihood (86.2% of time in **Fig 2**) of cost-effective acceptance of afatinib versus chemotherapy, similar to the China analyses. The afatinib strategy was not accepted to be cost-effective versus the erlotinib strategy in the present analysis and it is likely due to the difference in local pricing of TKIs in Hong Kong and China. The cost of erlotinib therapy was consistently lower than that of afatinib therapy in both regions, yet the cost difference between erlotinib and afatinib therapy was more substantial in Hong Kong than in China [21].

EGFR-testing guided afatinib was evaluated against empirical first-line chemotherapy in China [23] and EGFR-guided erlotinib was compared with empirical chemotherapy in South Korea [24]. Both analyses reported that the EGFR-mutation guided TKI was cost-effective when compared with empirical chemotherapy. The present analysis evaluated EGFR-guided use of 3 TKIs (afatinib, erlotinib and gefitinib), and empirical chemotherapy was also included for benchmarking. Comparison between EGFR-guided use of afatinib, erlotinib and gefitinib was lacking in the literature of health economic analyses, and our study provided findings to fill this research gap. The present findings showed consistent cost-effective acceptance of EGFR-mutation guided erlotinib and afatinib versus empirical chemotherapy, and found the EGFR-testing guided erlotinib to be preferred cost-effective strategy in Hong Kong.

Treatment of advanced NSCLC remains a challenge and therapeutic agents such as new generation of TKIs are clinically promising yet remarkably costly. The cost-effectiveness of testing on actionable target to guide NSCLC therapy has been examined on gene testing of anaplastic lymphoma kinase for crizotinib therapy in China, and T70M resistance for osimertinib therapy in Canada [25, 26]. The health economics analysis of TKI (such as erlotinib) for maintenance therapy had also been examined in UK [27]. The findings in literature suggested that the acceptance of these drug therapy for NSCLC are highly subjective to the region-specific cost of therapy and WTP threshold. Despite the routine recommendations on testing of EGFR mutation for NSCLC across countries [3, 28], region and heath-system specific cost-effectiveness analysis on the use of testing-guided therapy is highly warranted to inform healthcare policy decision makers on selection of new NSCLC treatment strategies.

The use of GDP-based WTP threshold provides information to guide healthcare policy-makers on value for money, yet a fixed ICER threshold is not the sole factor to decide the acceptance of a new intervention. Affordability and budget impact should also be considered together with cost-effectiveness findings in the decision-making process. The present findings mapped out the probabilities of empirical chemotherapy and EGFR mutation-guided TKIs to be accepted as cost-effective over a broad range of WTP for benchmarking in local and nearby regions.

There are some limitations in the present study. The model simplified real-life events of advanced NSCLC therapy. The SAEs of TKI and chemotherapy, with high incidence or with severe outcomes requiring medical therapy, were included. The impact of less serious adverse events were not fully represented. Both first-line chemotherapy and TKIs showed good tolerability when patients were provided with adequate supportive measures for adverse events, and treatment discontinuation due to SAEs was therefore not included in the present model. The present model examined chemotherapy and TKIs for the first-line treatment of advanced NSCLC, and the use of chemotherapy for maintenance treatment was not included. The use of maintenance pemetrexed in patients who did not progress after cisplatin-pemetrexed is a common clinical practice. The use of chemotherapy and TKIs for maintenance treatment warrant further cost-effectiveness evaluation. Publications written in English was selected in the literature search for model clinical inputs, and there were possible relevant findings reported in other language (such as Chinese language) not included in the present model. Also, the patients’ loss of productivity was not included and might therefore underestimate the impact of NSCLC treatment on indirect cost.

## Conclusions

EGFR mutation-guided use of afatinib, erlotinib and gefitinib appear to gain higher QALYs than empirical chemotherapy (without EGFR mutation testing). EGFR mutation-guided erlotinib seems to save cost and is likely to be the cost-effective strategy from the perspective of public healthcare provider of Hong Kong. The cost-effectiveness acceptance of EGFR mutation-guided afatinib is highly subject to the monthly cost of afatinib therapy.
